# Transcribing RNA polymerase III observed by electron cryomicroscopy

**DOI:** 10.1111/febs.13732

**Published:** 2016-04-28

**Authors:** Niklas A. Hoffmann, Arjen J. Jakobi, Matthias K. Vorländer, Carsten Sachse, Christoph W. Müller

**Affiliations:** ^1^ European Molecular Biology Laboratory (EMBL)Structural and Computational Biology UnitHeidelbergGermany

**Keywords:** electron cryomicroscopy, Pol III, RNA polymerase III, transcription, tRNA

## Abstract

Electron cryomicroscopy reconstructions of elongating RNA polymerase (Pol) III at 3.9 Å resolution and of unbound Pol III (apo Pol III) in two distinct conformations at 4.6 Å and 4.7 Å resolution allow the construction of complete atomic models of Pol III and provide new functional insights into the adaption of Pol III to fulfill its specific transcription tasks.

**Abbreviations**
apo Pol III: unbound RNA polymerase III
cryo‐EM: electron cryomicroscopy
pIC: preinitiation complex
Pol III: RNA polymerase III
Pol II: RNA polymerase II
Pol I: RNA polymerase I
RNAP: RNA polymerase

## Introduction

**Figure 1:**
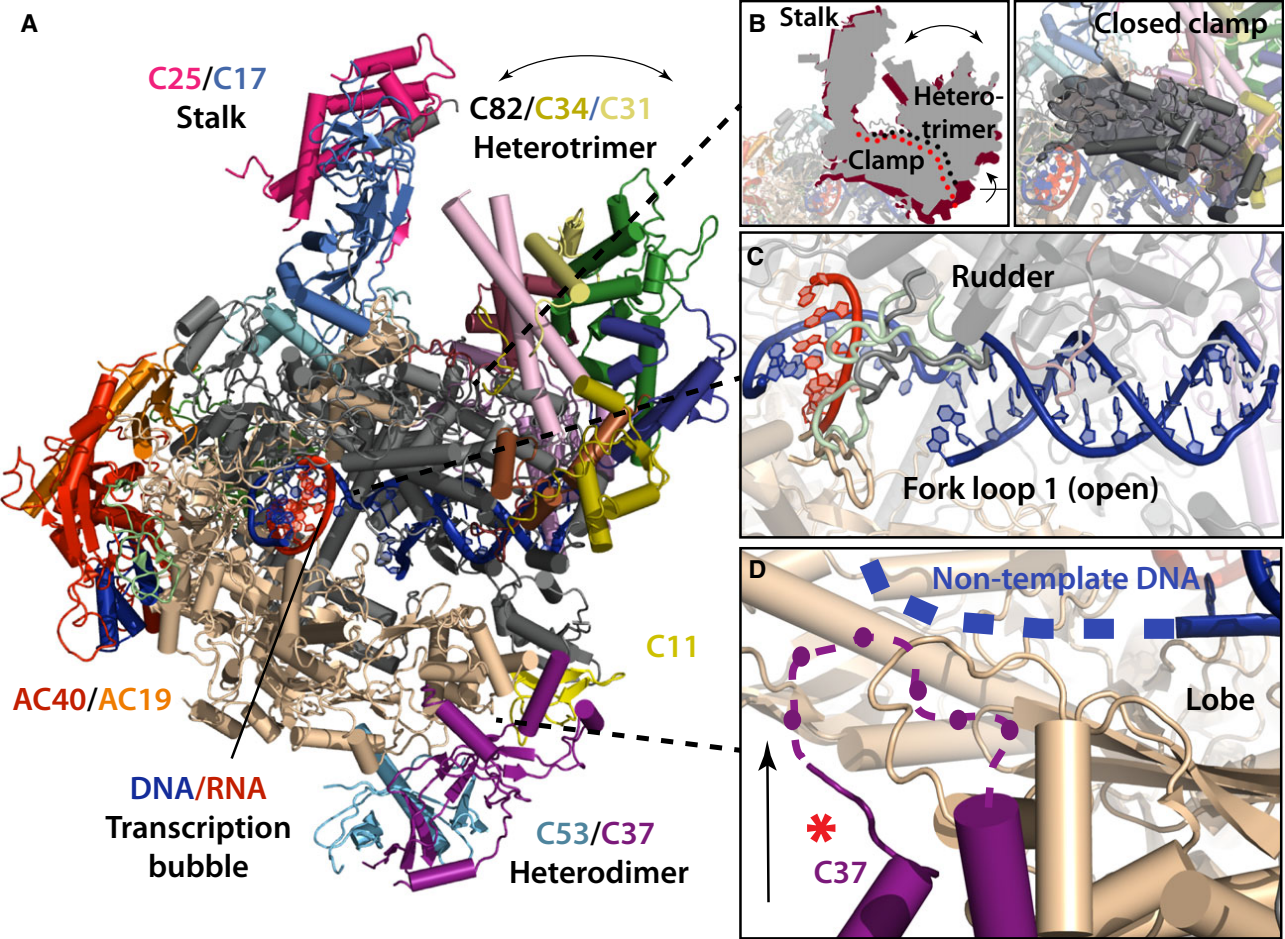
Structure and functional context of Pol III. (A) Structure of 17‐subunit elongating Pol III. The color code is according to Ref. 12. Peripheral subunits and subcomplexes are indicated. Helices are shown as cylinders. (B) Conformational flexibility of apo Pol III is displayed schematically (left panel). The red scheme represents the ‘closed clamp’ conformation and the gray scheme the ‘open clamp’ conformation. Dots indicate the clamp domain (red – closed clamp, black – open clamp), the panel on the right shows the clamp domain in ribbon representation. (C) DNA(blue)/RNA(red) duplex bound by elongating Pol III. Rudder and fork loop 1 of Pol III are displayed with larger tube radius for better visibility. The green loops show the alternating Pol II conformation of both elements. (D) Close proximity of subunit C37 (purple) to the nontemplate strand (blue). An extended C37 loop crossing the lobe domain (purple dashed line) positions C37 residues in close proximity to the tentative path of the nontemplate DNA strand. The red asterisk marks the position of the C37 residues important for accurate termination 25, 27.

RNA polymerases (RNAPs) are large macromolecular machines that transcribe RNA molecules from DNA templates. Bacteria and archaea contain one RNAP responsible for the entire RNA production. In eukaryotes, this task is divided among three RNAPs that transcribe the bulk of RNA. RNA polymerase I (Pol I) contains 14 subunits and transcribes ribosomal precursor RNA, whereas RNA polymerase II (Pol II) harbors 12 subunits and transcribes mainly mRNA and small, regulatory RNAs. Transcription of short, structured RNAs including all tRNA, U6 snRNA, and 5S rRNA is carried out by the largest eukaryotic RNAP, RNA polymerase III (Pol III) 1 containing 17 subunits with a total mass of 700 kDa. Despite this division of tasks, many basic mechanisms of transcription are conserved among the three eukaryotic RNAPs 2. Consequently, the first near‐atomic resolution structure of a eukaryotic RNAP, namely Pol II, was solved by X‐ray crystallography at the beginning of the millennium and provided the structural basis for rationalizing decades of research on RNAP function 3, 4, 5, 6. Nevertheless, adaptations among the different RNAPs accounting for their specific transcription profiles remained elusive and despite multiple efforts, it took an additional decade to solve the crystal structure of Pol I 7, 8. For Pol III, first structural insight was obtained from a low‐resolution electron cryomicroscopy (cryo‐EM) study of native unbound Pol III (apo Pol III) 9 that revealed its overall topology and the approximate positions of two Pol III‐specific subcomplexes using antibody‐labeling. Additional cryo‐EM studies on apo Pol III and on Pol III transcribing a DNA/RNA scaffold (elongating Pol III) further elucidated the topology and functionality of the enzyme 10, 11. Nevertheless, the limited resolution of the different cryo‐EM reconstructions restricted more accurate subunit positioning and additional mechanistic insights. The recent technological advances in electron microscopy provided a turning point, leading to the first near‐atomic resolution structures of apo Pol III and elongating Pol III 12. For the first time, Pol III‐specific transcription can now be studied at molecular detail allowing unprecedented insights into the structural adaptation of the Pol III machinery toward its biological function (Fig. 1). This is of special relevance in light of recent research that increasingly implicates misregulation of Pol III transcription in a number of diseases. Finally, the study completes the gallery of eukaryotic RNAPs and thus contributes to a broader view on transcription in general, thereby enabling new perspectives for research and clinical therapy.

## Atomic model building and refinement of Pol III using cryo‐EM maps

**Figure 2:**
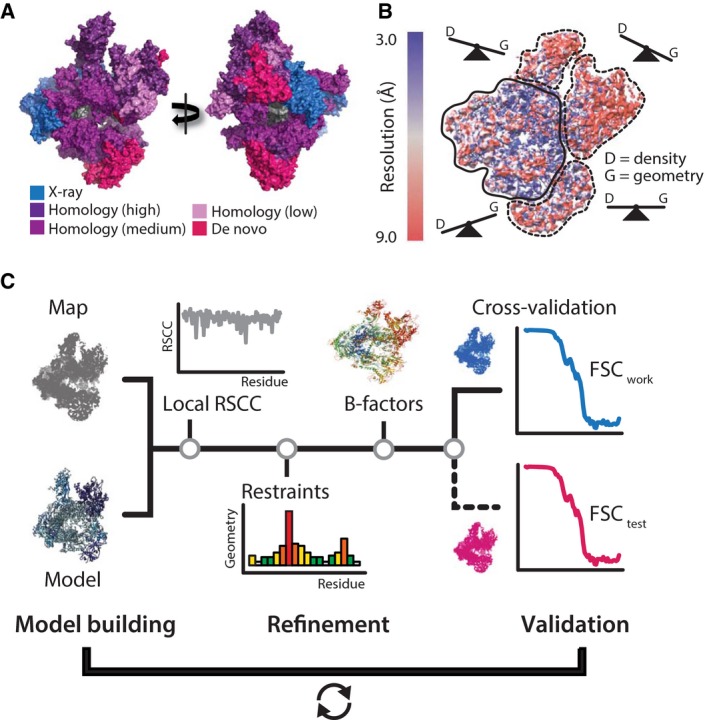
Outline of the model building and refinement workflow. (A) Sources of initial coordinate models mapped onto a surface representation of the Pol III complex. Model building was guided by available crystal structures, homology models with variable levels of confidence as indicated in the color legend, or models were built *de novo* where prior structural information was not available. (B) Schematic representation of locally optimized restraint weighting employed during refinement. Density and geometry restraints are weighted according to the local resolution in map sectors. (C) Schematic outline of the model refinement workflow. Models were built and subsequently refined against the map from all data by minimization of the map (real‐space correlation, RSCC) and restraint (model geometry) target. The refinement procedure was cross‐validated by using the agreement of the Fourier shell correlation (FSC) curves calculated between model and one half map (FSC_work_) and the half map not used for refinement (FSC_test_) as a criterion against overfitting.

Using single‐particle cryo‐EM, we determined three structures of Pol III from *Saccharomyces cerevisiae*, one in the elongating state at 3.9 Å resolution and two for distinct conformations of the apo enzyme at 4.6 Å and 4.7 Å resolution. Although these structures can be considered high resolution by cryo‐EM standards, such densities are still challenging for atomic interpretation and thus coordinate refinement is not routine. In order to build a complete atomic model, we focused our efforts on the 3.9 Å resolution map of the elongating Pol III (Fig. 1A). The most detailed density features were observed in the 10 subunit core complex, a region that contains most conserved subunits including the seven subunits shared with Pol I (five with Pol II). For these latter subunits, high‐ or intermediate‐resolution crystal structures were available 4, 7. Together with high‐quality density for the two large subunits, C160 and C128, building an atomic model of the Pol III core was therefore straightforward using interactive model building tools. However, much less structural information was available for the peripheral Pol III subunits, in particular, the C82/C34/C31 heterotrimer and the C53/C37 heterodimer (Fig. 2A).

As EM maps possess experimental amplitudes and phases, map quality is not dependent on model phases or iterative phase improvement and hence less prone to model bias. To assist chain tracing by empirical structural data, we used homolog structures and secondary structure predictions as references. Due to the known decay of amplitude contrast 13, EM maps require sharpening by a negative B‐factor to visualize high‐resolution detail. However, if resolution varies substantially throughout the map, a uniform sharpening factor can lead to lack of existing features in some parts and enhancement of noise in other parts of the map. Therefore, we generated a series of B‐factor sharpened maps to improve visibility of high‐resolution features and improve density connectivity in different map sectors. Once initial models had been generated and refined, we applied amplitude scaling derived from the atomic models to the map. This resulted in enhanced visibility of features and facilitated *de novo* building of poorly resolved regions.

Atomic coordinate refinement strives for the maximum agreement of the model with a density map in cryo‐EM or diffraction amplitudes in X‐ray crystallography while optimally weighting the preservation of known model geometry. In X‐ray crystallography, most existing protocols rely on reciprocal space refinement, which cannot account for resolution variation across the map as evident in the Pol III cryo‐EM maps. Real‐space refinement is better suited to deal with resolution differences as it is driven solely by local density features. To reduce complexity of the refinement, we first refined individual subunits separately against their respective map segments with restraint weights optimally balanced for the average local resolution of this map segment. For refinement of the entire 17 subunit Pol III complex, we then implemented local refinement weights that were scaled relative to the global refinement weights by a factor estimated from the ratio of local over the average global resolution. Hence, we effectively adjusted geometry and other external restraints to the variable confidence levels present in the map (Fig. 2B). This procedure improved both real‐space correlation and geometry statistics of the refined models and may represent a more general approach to take into account the resolution differences during atomic coordinate refinement using cryo‐EM maps. During the refinement geometry, violations were constantly monitored by evaluation of model geometry using Molprobity 14. We also implemented a conformation analysis based on virtual dihedrals (CaBLAM 15) as part of every refinement iteration that helped to diagnose problematic regions and redefine secondary structure restraints.

A general issue of low‐resolution coordinate refinement is the risk of overfitting as the observable‐to‐parameter ratio is poor. In X‐ray crystallography, a set of randomly chosen structure factors omitted during model building and refinement is used to cross‐validate the model (free R factor) 16. This approach is not applicable to EM data as individual Fourier coefficients cannot be considered independently and are strongly correlated 17. Therefore, we pursued an approach that performs the refinement using the complete map as target and testing the validity of the chosen refinement parameters by subsequent assessment against two independent half‐maps, i.e., one work and one test map. After perturbing the model by random atom displacement followed by re‐refinement against the work map (Fig. 2C), overfitting of the model is assessed by Fourier shell correlation against the independent test map. This way, structure refinement against the complete map significantly improves the accuracy of the model while it robustly safeguards against overfitting.

## The Pol III structures provide functional insights into Pol III‐specific transcription

The final model of Pol III comprises all 17 subunits and shows an overall conserved architecture when compared to Pol I and Pol II (Fig. 1A). The cleft is narrower than previously observed for other eukaryotic RNAPs, and several subunits of the core show uncharacterized insertions to date. The structure of Pol III also provides a more detailed view on the Pol III‐specific subcomplexes C53/C37 and C82/C34/C31, which allows their better functional characterization as discussed below. A striking discovery of the study is the conformational flexibility observed in apo Pol III. One conformation resembles the elongating Pol III state and was termed ‘closed clamp state’ after the moving clamp domain of the largest Pol III subunit C160, whereas the second conformation was termed ‘open clamp state’ and contains a more open clamp (Fig. 1B). Interestingly, open and closed clamp conformations are also present in archaeal Pol and Pol II, and in both systems, they are associated with the stalk, a dimeric subcomplex that protrudes from the RNAP core and recruits initiation factors 5, 18, 19, 20. In Pol III, one connection between stalk and clamp domain is formed by subunit C82, part of the Pol III heterotrimer, that extends over the clamp domain toward incoming downstream DNA. Furthermore, a loop termed C82 ‘cleft loop’ protrudes the clamp to reach an enclosed cavity close to the active site. Interestingly, the cleft loop contains two arginines positioned in close proximity to the DNA in a model of the preinitiation complex (pIC). A transition from the open clamp state to the closed clamp state could aid promoter melting of the closed DNA duplex during transcription initiation as seen in the bacterial system 21. Moreover, the flexible clamp could not only lead to relocation of C34 in Pol III, but presumably also to the related general transcription factor TFIIE in Pol II, thereby assisting the melting process. In this scenario, the stalk could play a central role in determining the clamp state and could relay the clamp transition through its association with additional initiation factors in a pIC.

The structure of elongating Pol III shows a tightly enclosed downstream DNA entering the cleft. Two elements of the large subunit C160 on the one side and subunits ABC27 and C82 on the other side enclose the incoming DNA. In addition, a proline‐containing loop of ABC27 inserts into the minor groove, thus threading the DNA toward the active site during transcription. A threading function for ABC27 has also been proposed in the Pol II system, but no strong enclosure similar to Pol III has been observed 5, 18. Surprisingly, the DNA/RNA duplex at the active site is only loosely associated with the Pol III enzyme (Fig. 1C). The fork loop 1 is in an open position, and the rudder is not as closely oriented toward the duplex as in Pol II. Notably, the tight enclosure of the transcription bubble in the active site of Pol II is presumably one main reason for its high processivity 22. In contrast, Pol III synthesizes shorter transcripts compared to Pol II and Pol I, and Pol III's unique abilities like ‘facilitated reinitiation’ and specific termination might benefit from such variations, even at the expense of speed and processivity. Additionally, several Pol III subunits influence and presumably interfere with transcription during the transcription cycle, which would require a more accessible and thus loosely associated DNA/RNA duplex 23, 24, 25.

Another major discovery of this study is the association and structure of the C53/C37 heterodimer on the lobe of Pol III, especially the conformation of subunit C37. Previous studies demonstrated the importance of C37 in transcription termination of Pol III, which unlike any other eukaryotic Pols only requires a stretch of 5–7 thymidines in the nontemplate DNA strand to terminate transcription 26. Our structure demonstrates how C37 positions five amino acid residues shown to be important for specific termination 25, 27, 28 in close proximity to the nontemplate strand (Fig. 1D). Binding of these residues to the stretch of thymidine in the nontemplate strand could provide the structural basis for transcription termination.

## Emerging relevance of Pol III as a potential drug target

Transcription by Pol III is a highly controlled process and under the influence of both positive and negative regulators to balance the need for cellular growth and proliferation on the one hand and metabolic efficiency on the other hand. Prominent proto‐oncogenes, such as Ras/ERK, PI3K, TORC1, and c‐Myc, activate Pol III transcription, whereas tumor suppressors, such as Rb protein, p53, and PTEN, act as repressors 29.

A factor of particular importance in Pol III regulation is Maf1, a global repressor of Pol III conserved from yeast to man 30. Maf1 represses Pol III under a variety of stress conditions and integrates signals from different pathways 31. In *S. cerevisiae*, Maf1 represses transcription by binding to Pol III as well as to the TFIIIB component Brf1, thereby preventing the formation of a pIC 32. Phosphorylation of Maf1 at several sites not only regulates its activity by preventing its nuclear import but also Maf1 binding to Pol III. Maf1 activity is regulated by TORC1, which is also an upstream regulator of the two protein kinases Kns1 and Mck1. In cooperation with C11, both kinases downregulate Pol III activity by phosphorylation of C53 33.

Not surprisingly, misregulation of Pol III activity has been implicated in a number of diseases and has long been known to be a feature of many tumors 34. Recent evidence also suggests that an increase in Pol III transcription does not merely represent an adaptation of tumor cell metabolism to increased growth rates but is essential for the transformation process 35, highlighting the importance of increased Pol III activity in tumorigenesis. Recently, a more specific TFIIIB related link to tumorigenesis was characterized in Brf2, which is a vertebrate‐specific Brf1 homolog that functions as a redox‐sensor, and was shown to be highly active in breast and lung cancer 36. Pol III transcriptional activity was also shown to influence cytokine secretion and phagocytosis in macrophages, which links Pol III to immune responses 37. Finally, Pol III malfunction has also been linked to a number of neurogenetic disorders that are not fully understood in their pathophysiology, but all feature severe developmental impairment, hypomyelation, and progressive neurodegeneration 38.

**Figure 3:**
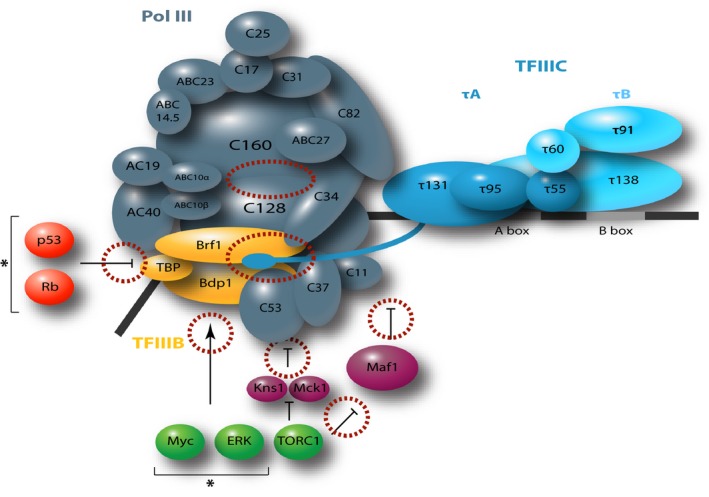
Pol III preinitiation complex (pIC) and its regulatory factors as potential drug targets. Scheme of the Pol III pIC with Pol III depicted in gray, TFIIIB in yellow, and TFIIIC in blue. DNA is depicted as black line. Subunits are named according to yeast nomenclature as given in Ref. 12 and in parentheses in Table 1. Positioning of the subunit TFIIIB is based on biochemical evidence and cross‐linking studies that describe interactions of Brf1 with C34 and τ131 44 and of Bdp1 close to the active site 49. Interaction of τ131 with Brf1 is schematically shown by the extension of τ131 toward TFIIIB and Pol III. Maf1 and the kinases, Kns1 and Mck1, are depicted in purple. Other regulatory factors and their reported role in transcriptional regulation of Pol III are indicated in red (inhibiting) and green (stimulating) circles. Factors marked with an asterisk are only present in metazoa. The dotted red circles mark potential drug target interfaces in the Pol III active site and in interfaces within the pIC or with regulatory factors.

Consequently, there is an increasing interest in pharmacological interference with Pol III transcription. Two strategies are conceivable for targeting Pol III with small molecules: one strategy is aimed at the inhibition of Pol III's enzymatic activity; the other strategy targets the assembly and regulation of the Pol III transcriptional machinery at the level of the pIC (Fig. 3). Natural compounds that specifically inhibit RNAP transcription exist, including thiolutin, α‐amanitin, and tagetitoxin. Thiolutin is a strong inhibitor of all three eukaryotic RNAPs 39, α‐amanitin strongly inhibits Pol II, is inactive against Pol I, and partially active against Pol III 40, whereas tagetitoxin strongly inhibits Pol III 41. In Pol II, α‐amanitin was shown to stabilize a translocation intermediate conformation 42. Interestingly, in Pol I and Pol III, the varying sensitivity against α‐amanitin correlates with different levels of occupancy of the TFIIS‐like C‐terminal domains of Pol I subunit A12.2 and Pol III subunit C11, which unlike TFIIS in Pol II are both part of the core enzymes. In Pol I, the C‐terminal domain of subunit A12.2 is stably associated with the core in close proximity to the active site and overlaps with the α‐amanitin binding site observed in Pol II consistent with the complete insensitivity of Pol I toward α‐amanitin. In Pol III, the TFIIS‐like C‐terminal domain of subunit C11 is less stably associated with the enzyme, but instead is only transiently recruited to the same site and indeed, Pol III shows an intermediate sensitivity for α‐amanitin. Apart from naturally existing inhibitors, a first approach to synthetically design compounds against Pol III yielded promising lead compounds, including ML‐60218 which shows strong inhibition of *S. cerevisiae, Candida albicans* and human Pol III 43. Despite the strong similarities between the eukaryotic RNAPs, it is possible to specifically inhibit transcription of one polymerase, although achieving selective inhibition of one RNAP while maintaining complete insensitivity of the other RNAPs remains a challenge.

The problem of finding specific inhibitors for only one of the three eukaryotic RNAPs can be surpassed by targeting the recruitment machinery of Pol III, which comprises specific and accessible interaction surfaces compared to the more conserved Pol III enzyme. Consequently, binding pockets and surfaces between Pol III and its specific transcription factors are potential drug targets that generally downregulate Pol III transcription. Examples include Pol III–TFIIIB interactions, such as the C34–Brf1 interface 44, 45 or the reported C37–Bdp1 interaction 27, Pol III–TFIIIC interactions 27, and ultimately TFIIIB–TFIIIC interactions 46. Maf1, but also protein kinases such as Kns1 and Mck1 that function downstream of TORC1 regulate and orchestrate Pol III activity and are thus interesting targets for Pol III‐specific transcriptional modulation. Finally, Pol III transcription is embedded in a large regulatory network where upstream and downstream effectors represent additional targets for therapeutic intervention. Therefore, structural and functional insights from model organisms, such as yeast, can help to better understand the human Pol III system.

ltimately, a more detailed understanding of the human Pol III system is especially relevant as many of the direct interactions of human proto‐oncogenes and tumor suppressors with the Pol III transcription machinery are interesting potential drug targets that will have to be explored in the future (Fig. 3). Despite the overall strong conservation of the core enzymes between yeast and human 47, several human Pol III subunits show significant differences reflected in a low sequence identity score, while the Pol III‐specific subunits of the general transcription factors TFIIIB and TFIIIC are even less conserved (Table 1). The better characterization of human Pol III‐specific adaptations, for instance the much larger C37 human ortholog HsRpc5 which contains an extended C‐terminal domain or the role of different isoforms of TFIIIB subunits Bdp1 and the Brf1‐related isoform Brf2 that functions in a more specialized cellular context 36, will help in the development of novel medical approaches and will prove essential in future drug development.

**Table 1:** Comparison of Pol III subunits in yeast and human

Yeast			Human			% Identity^b^
Subunit^a^	Gene	m (kDa)	Subunit^a^	Gene	m (kDa)	
*RNA polymerase III*						
Sc**RPC1** (C160)	RPO31	162.3	Hs**RPC1** (RPC155)	POLR3A	155.6	49.2 (723)
Sc**RPC2** (C128)	RET1	129.5	Hs**RPC2**	POLR3B	127.8	61.1 (708)
Sc**RPC3** (C82)	RPC82	74.0	Hs**RPC3** (RPC62)	POLR3C	60.6	15.7 (110)
Sc**RPC4** (C53)	RPC53	46.7	Hs**RPC4** (RPC53)	POLR3D	44.4	18.1 (85)
Sc**RPC5** (C37)	RPC37	32.1	Hs**RPC5** (C37)	POLR3E	79.9	7 (54)
Sc**RPC6** (C34)	RPC34	36.1	Hs**RPC6** (RPC39)	POLR3F	35.7	23.3 (78)
Sc**RPC7** (C31)	RPC31	27.7	Hs**RPC7** (RPC32)	POLR3G	25.9	23.6 (61)
Sc**RPC8** (C25)	RPC25	24.3	Hs**RPC8**	POLR3H	22.9	41.9 (91)
Sc**RPC9** (C17)	RPC17	18.6	Hs**RPC9** (CGRP‐RC)	CRCP	16.9	23.8 (44)
Sc**RPC10** (C11)	RPC11	12.5	Hs**RPC10** (RPC11)	POLR3K	12.3	50 (22)
Sc**RPAC1** (AC40)	RPC40	37.7	Hs**RPAC1** (RPA5)	POLR1C	39.3	43 (150)
Sc**RPAC2** (AC19)	RPC19	16.1	Hs**RPAC2** (RPA9)	POLR1D	15.2	35.1 (54)
Sc**RPABC1** (ABC27)	RPB5	25.1	Hs**RPABC1** (RPB5)	POLR2E	24.6	41.3 (90)
Sc**RPABC2** (ABC23)	RPO26	17.9	Hs**RPABC2** (RPB6)	POLR2F	14.5	49.4 (72)
Sc**RPABC3** (ABC14.5)	RPB8	16.5	Hs**RPABC3** (RPB8)	POLR2H	17.1	31.9 (50)
Sc**RPABC4** (ABC10α)	RPC10	7.7	Hs**RPABC4** (RPB7.0)	POLR2K	7.0	31.4 (22)
Sc**RPABC5** (ABC10β)	RPB10	8.3	Hs**RPABC5** (RPB10)	POLR2L	7.6	71.4 (50)
*TFIIIB*						
Sc**TBP**	SPT15	27.0	Hs**TBP**	TBP	37.7	45.3 (154)
Sc**Brf1**	BRF1	66.9	Hs**Brf1**	BRF1^c^	73.8	24.4 (183)
Sc**Bdp1**	BDP1	67.7	Hs**Bdp1**	BDP1	293.9	3.9 (108)
*TFIIIC*						
**Tfc1** (τ95)	TFC1	73.5	**TFIIIC63**	GTF3C5	59.6	15.8 (111)
**Tfc3** (τ138)	TFC3	132.1	**TFIIIC220**	GTF3C1	238.9	8.1 (185)
**Tfc4** (τ131)	TFC4	120.2	**TFIIIC102**	GTF3C3	101.3	19.7 (215)
**Tfc6** (τ91)	TFC6	74.7	**TFIIIC110**	GTF3C2	100.7	10.8 (113)
**Tfc7** (τ55)	TFC7	49.2	**TFIIIC35**	GTF3C6	24.1	6.2 (31)
**Tfc8** (τ60)	TFC8	67.7	**TFIIIC90**	GTF3C4	92.0	11.2 (99)

a Pol III subunit nomenclature is based on the nomenclature suggested in Ref. 47. Alternative names of *Saccharomyces cerevisiae* and *Homo sapiens* specific subunits are given in brackets.

b Values were calculated with the Uniprot Align tool. When multiple isoforms were present, the most common isoform based on Uniprot was used for the alignment. The number of identical amino acids is given in brackets.

c Alignment with human Brf1 isoform Brf2: m (kDa) – 46.5 kDa; % Identity – 10.9% (72).

## Conclusion and Perspectives

The cryo‐EM structures of apo Pol III and elongating Pol III provide the first atomic models of this enzyme. The observed clamp flexibility relayed by the stalk, the discovered loose association of the DNA/RNA duplex in the active site, and first structural insight into Pol III‐specific transcription termination are key discoveries of this study. However, many open questions still remain. Some parts of the Pol III‐specific subunits in the C53/C37 heterodimer and C82/C34/C31 heterotrimer are highly mobile and are not resolved in the structures. For example, the flexible N‐terminal extension of C53 is not visible in the cryo‐EM structure although it has been cross‐linked to the active site and the stalk 23, 27. Similarly, the flexible C‐terminus of C31 is disordered. The N‐terminal two winged helix domains of C34, important for initiation and the interaction with Brf1 44, 48, are also mobile and have not been included in the final model. Furthermore, the conformational flexibility of the clamp, the heterotrimer and the stalk, and its biological role in transcription initiation and regulation need to be further explored. The large interaction interface around the lobe region of subunit C128 also involves subunits C34 and C37, and likely TFIIIB subunits Brf1 and Bdp1 49. It thereby links Pol III with TFIIIB and presumably allows the direct transition from termination to reinitiation on the same gene in a process known as ‘facilitated reinitiation’ 50. Finally, high‐resolution structures of a Maf1–Pol III complex, a Pol III pIC and complexes between regulatory factors and the Pol III‐specific general transcription factors, TFIIIB and TFIIIC, will allow reconstituting a full Pol III transcription cycle, but will also contribute in directing future drug design efforts.

## Author contributions

All authors contributed to writing the manuscript.
